# Application of fractional flow reserve and optical coherence tomography examinations in a patient presenting with recurrent angina: a case report

**DOI:** 10.1186/s13256-015-0664-y

**Published:** 2015-08-27

**Authors:** Neng Dai, ShaoTang Lu, XianKai Li, YaWei Xu, WeiMing Li

**Affiliations:** 1 Cardiology Department, Tenth People’s Hospital, Tongji University School of Medicine, Shanghai, China; 2 Cardiology Department, DongTai People’s Hospital, DongTai, Jiangsu Province China

**Keywords:** Coronary artery disease, Fractional flow reserve, Optical coherence tomography, Plaque rupture

## Abstract

**Introduction:**

We present the different roles of fractional flow reserve and optical coherence tomography in guiding treatment in a patient with recurrent chest pain.

**Case presentation:**

A 66-year-old Chinese woman presented to our department for the third time for her recurrent chest pain. Her physical examination was unremarkable; her previous two angiography examinations indicated that there was a stenosis of 50 to 70% in her proximal left anterior descending coronary artery. Optimal medical therapy was applied, but her symptoms did not disappear. Coronary angiography was conducted again after admission, accompanied by fractional flow reserve and optical coherence tomography. A lesion of 50 to 70% in her left anterior descending coronary artery was detected in an angiogram as before; her fractional flow reserve measure was a negative result of 0.88. However, a plaque rupture was found at the location of the lesion in the optical coherence tomography imaging. A stent was implanted in her left anterior descending coronary artery; she made no complaint of chest pain during follow-up of 1.5 years after her discharge.

**Conclusions:**

Fractional flow reserve is considered the “gold standard” to detect ischemia-causing lesions and provide hemodynamic information of a stenosis. However, lack of structural information of a stenosis limits the application of fractional flow reserve and coronary pressure may lie sometimes. We should choose the best strategy for patients according to different examinations and patients’ symptoms, never a single test.

## Introduction

Angina is the most common symptom for patients with coronary artery disease (CAD) when the flow of oxygen-rich blood to the heart muscle is reduced by atherosclerotic plaque [1]. Fractional flow reserve (FFR) is now considered the gold standard in determining severity of myocardial ischemia. Current guidelines state that decision making about coronary revascularization should be guided by myocardial ischemia [2], and revascularization procedures performed in patients with documented ischemia reduce total mortality through reduction of ischemic burden [3, 4]. Optical coherence tomography (OCT) is another commonly used intravascular examination in clinical practice, which uses near-infrared light to create images. By presenting a signal-rich layer nearest the lumen, poor signal in middle layer, and rich signal surrounding the signal poor layer of the media, respectively, three layers of the coronary artery wall can be discriminated in most cases [5]. In addition, OCT is helpful in identifying different types of coronary plaques, such as fibrous, fibrocalcific, and lipid plaques, according to their distinctive signals. Furthermore, OCT can detect plaque rupture, erosion, intracoronary thrombus, thin-cap fibroatheroma, and calcified nodule [6], which thus is a promising imaging modality for guiding percutaneous coronary intervention (PCI) therapy, though not many studies about OCT in guiding PCI and assessing its impact on cardiovascular outcomes are available at present.

Here we report a case with a negative FFR measurement but positive OCT result, a stent implant was conducted despite the negative FFR and follow-up revealed that the patient benefitted from this strategy.

## Case presentation

A 66-year-old Chinese woman presented to our department for the third time for her recurrent chest pain. No fever, cough, dysphagia, hemoptysis or shortness of breath were reported. She denied history of hypertension, diabetes, hypercholesterolemia and cigarette smoking. Her father had a heart attack at the age of 70 years. A physical examination was unremarkable. An electrocardiogram revealed non-specific T-wave changes, but laboratory analysis demonstrated elevated serum troponin (0.063ng/ml, normal range: <0.014ng/ml) and creatine kinase MB (CK-MB; 8.97ng/ml, normal range: <5ng/ml).

**Fig. 1:**
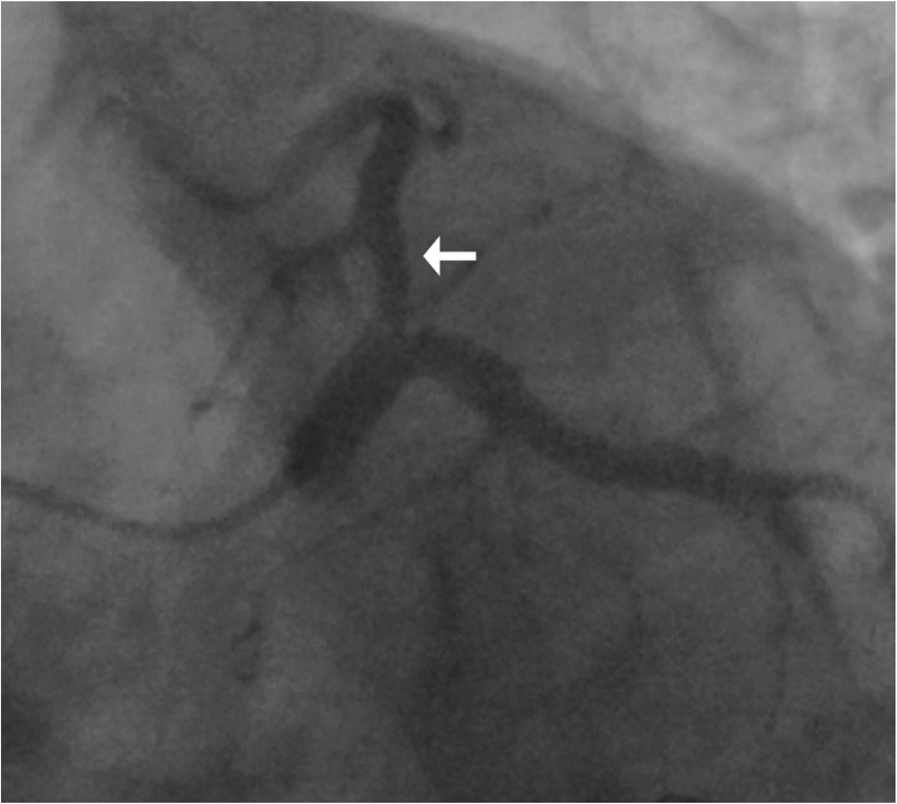
Coronary angiogram of the patient on her first admission. The angiogram was achieved on a spider view (left anterior oblique 45°, caudal 20°). The *white arrow* indicates that there is an intermediate lesion (50–70%) in proximal left anterior descending coronary artery

**Fig. 2:**
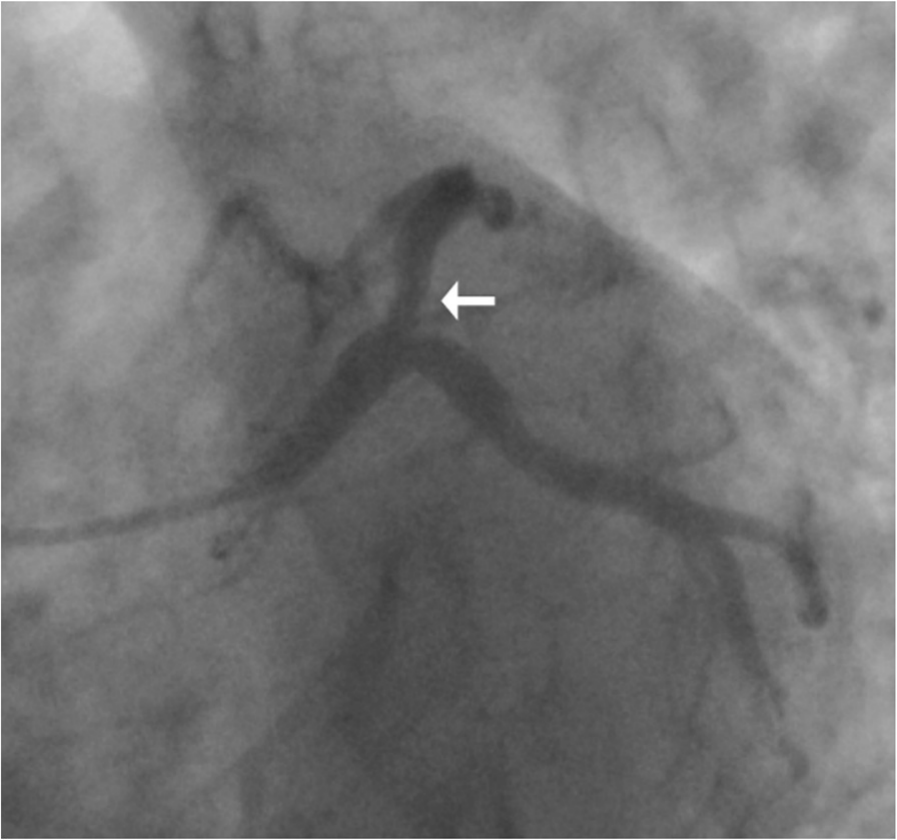
Coronary angiogram of the patient on her second admission. The angiogram was achieved on a spider view (left anterior oblique 44°, caudal 22°). The *white arrow* indicates that there is an intermediate lesion (50–70%) in proximal left anterior descending coronary artery

She had had two coronary angiography examinations on 31 January 2012 and 17 January 2013, the results showed that there was a 50 to 70% stenosis in her proximal left anterior descending (LAD) coronary artery (Figs. 1 and 2). A FFR examination was suggested during her second angiography to testify whether there was a lesion-induced myocardial ischemia but was declined. She was discharged with optimal medical therapy: 100mg/day, atorvastatin 20mg/day, metoprolol 50mg/day, and valsartan 80mg/day.

**Fig. 3:**
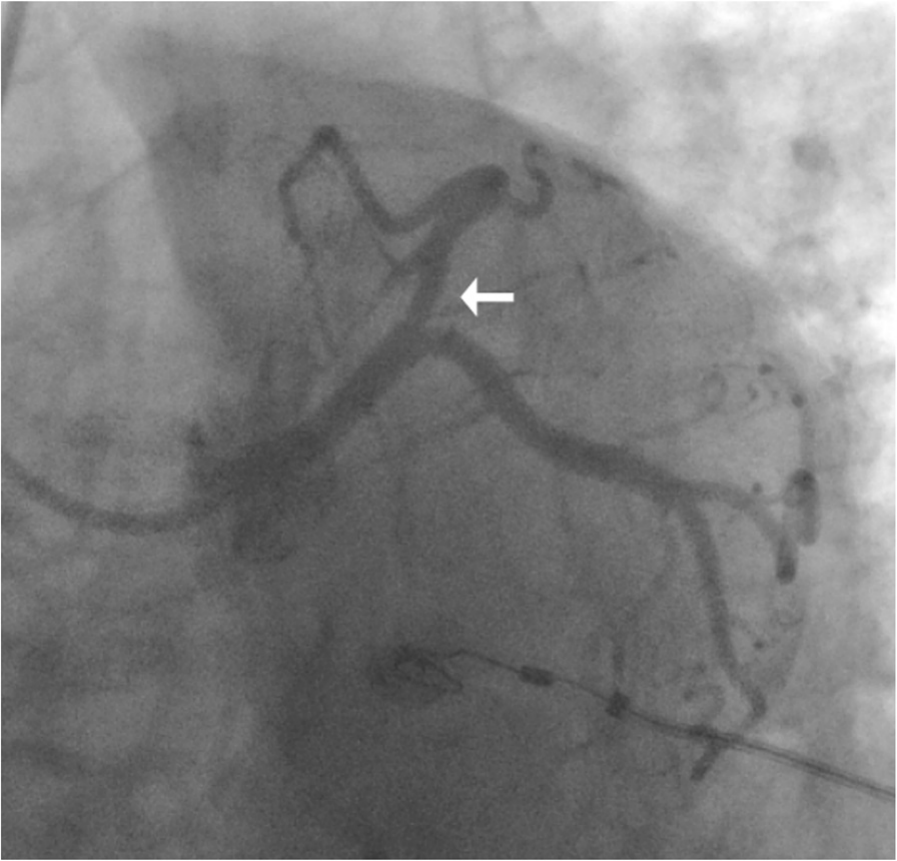
Coronary angiogram of the patient on her third admission. The angiogram was achieved on a spider view (left anterior oblique 47°, caudal 30°). The *white arrow* indicates that there is an intermediate lesion (50–70%) in proximal left anterior descending coronary artery

**Fig. 4:**
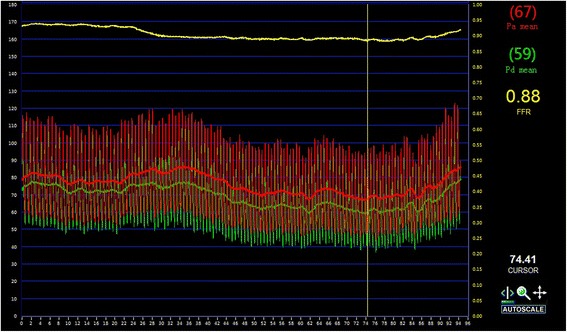
Fractional flow reserve measurement for left anterior descending coronary artery of the patient. The fractional flow reserve of left anterior descending coronary artery measurement was 0.88, indicating that no significant myocardial ischemia was induced by the lesion in left anterior descending coronary artery

**Fig. 5:**
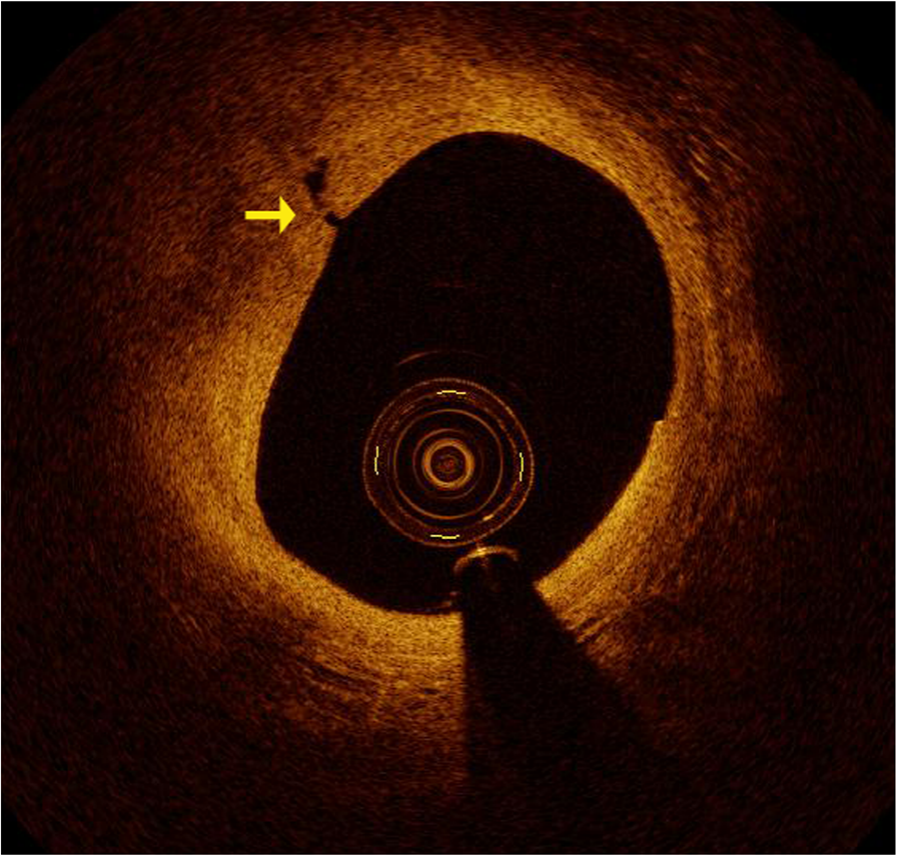
Optical coherence tomography imaging for the intermediate lesion in left anterior descending coronary artery. Optical coherence tomography imaging revealed a rupture (*yellow arrow*) in the intermediate stenosis on left anterior descending coronary artery

The angiography was conducted again after her third admission. A lesion of 50 to 70% in her LAD was detected in an angiogram (Fig. 3) as in previous results. In addition to angiography, FFR was also measured with a coronary guidewire (PressureWire Aeris, St. Jude Medical) during intravenous adenosine-induced hyperemia to assess the hemodynamic severity of each indicated stenosis. The FFR measured was 0.88 (Fig. 4), indicating that there was no significant myocardial ischemia induced by the LAD lesion. OCT imaging was then performed at a pullback speed (20mm/second) just under contrast flushing with a C7 Dragonfly catheter (St. Jude Medical), which characterized the lesion as an eccentric lipid plaque with a rupture (Fig. 5), the minimal luminal area was 4.9mm^2^. A stent was implanted in her LAD; she made no complaint of chest pain during follow-up for 1.5 years after her discharge.

## Conclusions

FFR is now widely accepted as the “gold standard” to detect ischemia-causing lesions and provide hemodynamic information of a stenosis. The prospective multicenter Fractional Flow Reserve versus Angiography for Multivessel Evaluation (FAME) trial revealed that FFR guidance in lesions with FFR ≤0.8 was associated with a 28% lower rate of major adverse clinical events compared to the previous gold standard non-FFR-guided angiography procedure [7]. Building upon these results, FAME II documented a significant reduction in downstream major adverse clinical events for those who underwent coronary revascularization for FFR-positive lesions versus optimal medical therapy [3]. The totality of data published thus far support ischemia, and FFR-guided revascularization in particular, over angiography guided revascularization. However, lack of structural information of a stenosis limits the application of FFR and coronary pressure may lie sometimes. As recently as 15 years ago, doctors thought that heart attacks occurred when plaques gradually built up until the arteries were too narrow to allow blood to flow to the heart. The majority of people who have a heart attack never have angina or other symptoms, however. Doctors now know that the majority of heart attacks occur when the artery is only slightly narrowed with culprit plaques that rupture. In addition, some plaques are less likely than others to rupture; these plaques are called vulnerable plaques. Several characteristics of vulnerable plaques have been identified by histology, including a large lipid pool, thin fibrous caps (<65μm), and activated macrophages [8, 9]. OCT can detect the vulnerable plaques or the ruptured ones because of its high resolution, which can add to functional examinations such as FFR, and thus have an impact on decisions regarding culprit lesion intervention. However, in a recent meta-analysis, D’Ascenzo et al. [10] found that OCT only had moderate diagnostic accuracy for detection of hemodynamically significant lesions defined by FFR, and the sensitivity and specificity of minimal luminal area (MLA) or of minimal luminal diameter (MLD) defined in OCT were inadequate to confidently guide revascularization. In intermediate lesions, although OCT has potential benefit as an adjunct examination in which the anatomy and composition of plaques of uncertain severity or morphology are evaluated, no recommendation should be given about OCT dimension and need for revascularization without a functional assessment such as FFR.

In this case, we presented a patient with an intermediate stenosis on coronary angiography and an FFR measurement that identified the lesion as a non-culprit one; however, OCT imaging detected a rupture of the plaque, which may be the reason for the angina she experienced and a potential factor leading to a heart attack. In this patient, optimal medical therapy had been administrated, but her symptoms and rupture continued to exist, which placed her at a high risk of rupturing again. We could have chosen a strategy of sequential medical treatment and clinical follow-up, but we conducted PCI instead for safety and compliance. In summary, we should choose the best strategy for patients according to different examinations and patients’ symptoms, never a single test. An examination which can combine the structural and functional assessments for coronary stenoses is strongly needed in the future.

## Consent

Written informed consent was obtained from the patient for publication of this case report and accompanying images. A copy of the written consent is available for review by the Editor-in-Chief of this journal.

## Abbreviations

CAD: Coronary artery disease
FAME: Fractional Flow Reserve versus Angiography for Multivessel Evaluation
FFR: Fractional flow reserve
LAD: Left anterior descending
OCT: Optical coherence tomography
PCI: Percutaneous coronary intervention
